# A Review of Topological Data Analysis and Topological Deep Learning in Molecular Sciences

**DOI:** 10.1021/acs.jcim.5c02266

**Published:** 2025-11-14

**Authors:** JunJie Wee, Jian Jiang

**Affiliations:** † Department of Mathematics, 3078 Michigan State University, East Lansing, Michigan 48824, United States; ‡ Research Center of Nonlinear Science, School of Mathematics and Statistics, Wuhan Textile University, Wuhan 430200, P.R. China

## Abstract

Topological data analysis (TDA) has emerged as a powerful framework for extracting robust, multiscale, and interpretable features from complex molecular data for artificial intelligence (AI) modeling and topological deep learning (TDL). This review provides a comprehensive overview of the development, methodologies, and applications of TDA in molecular sciences. We trace the evolution of TDA from early qualitative tools to advanced quantitative and predictive models, highlighting innovations such as persistent homology, persistent Laplacians, and topological machine learning. The paper explores TDA’s transformative impact across diverse domains, including biomolecular stability, protein–ligand interactions, drug discovery, materials science, topological sequence analysis, and viral evolution. Special attention is paid to recent advances in integrating TDA with machine learning and AI, enabling breakthroughs in protein engineering, solubility, and toxicity prediction, and the discovery of novel materials and therapeutics. We also discuss the limitations of current TDA approaches and outline future directions, including the integration of TDA with advanced AI models and the development of new topological invariants. This review aims to serve as a foundational reference for researchers seeking to harness the power of topology in molecular sciences.

## Graphical abstract

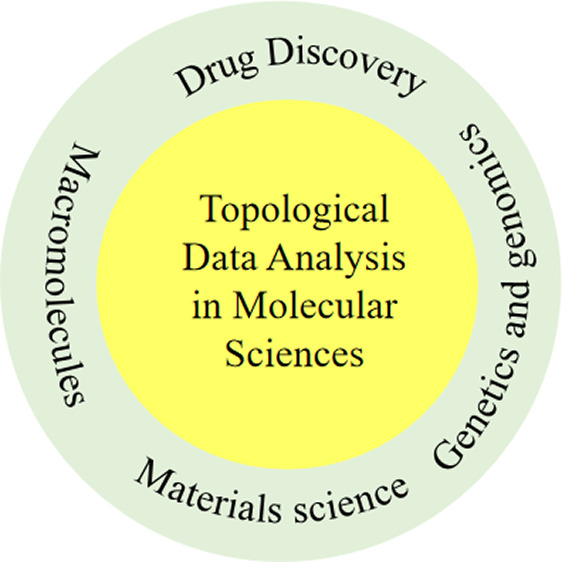

## 1 Introduction

Molecular science is a broad field of scientific inquiry that focuses on studying the structure, properties, and interactions of molecules, which are the fundamental building blocks of matter, materials, chemicals, and life. It encompasses various disciplines, including chemistry, physics, biology, materials science, and nanotechnology. Molecular science seeks to understand the behavior of molecules at the atomic and molecular levels and how this behavior impacts macroscopic properties and phenomena. Overall, molecular science is a multidisciplinary field that spans multiple disciplines and explores the fundamental principles governing the behavior of molecules. It plays a crucial role in advancing scientific knowledge and technological innovation in areas such as medicine, materials science, energy, and environmental science.

The basic method for the study of molecular science is experimentation, which has led to abundant data in the past few decades, facilitating data-driven discovery. However, data in molecular science are notoriously challenging to analyze due to their complexity, high dimensionality, multiscale, high-order interactions, nonlinear relations, etc. For example, single-cell RNA sequence (sc-RNA seq) data are intrinsically highly dimensional, involving tens of thousands of dimensions. Human genomic data are excessively large, containing about 3 billion bases. Macromolecular structures are intricate and diverse. Molecular interactions are high-order and complex due to many-body effects and a wide range of interactions, from covalent bonds, noncovalent bonds, hydrogen bonds, van der Waals, π–π stacking, electrostatic, to hydrophobic interactions. Conventional approaches for tackling molecular data include descriptive, inferential, spatial, temporal, physical, Fourier, and statistical analyses. Many challenges for conventional molecular data analyses can be effectively addressed by topological data analysis (TDA),^22^ an emerging field in data science and a new research frontier in applied mathematics.

A prominent technique in TDA is persistent homology. As a basic technique, it combines concepts from algebraic topology and multiscale analysis to analyze data.^57,127,200^ It detects complex topological invariants and patterns in data at various scales, which are not easily discernible with traditional geometric and statistical techniques. Topological invariants provide explainable representations of data^184–186^ that cannot be obtained from other alternative methods. Topological invariants are often represented as persistence barcodes,^66^ persistence images,^2,159^ persistence landscapes,^13,84^ and persistence surfaces.^2^

However, persistent homology has several limitations, including the lack of localization, being restricted to point cloud data, and the inability to represent nontopological information.^148^ In recent years, much effort has been devoted to addressing these challenges by Wei and his team members, motivated by challenges in molecular sciences. In order to incorporate additional information in simplicial complexes, element-specific persistent homology (ESPH),^20^ multilevel persistent homology,^14^ atom-specific persistent homology,^12^ electrostatic persistence,^14^ and weighted persistent homology^4,65,109^ were proposed and applied in biomolecular data analysis. Additionally, persistent homology or TDA in general inherently simplifies the data. As such, it may not be suitable for simple data, where the TDA simplification may lead to the loss of essential information. Most data in molecular sciences are very complex and require a reduction in their analysis. Over the years, many developments in TDA algorithms have been inspired by the needs and challenges in molecular sciences.

Persistent cohomology was proposed to handle heterogeneous information.^21^ Wei and his co-workers (i.e., Wei team) introduced persistent spectral theory^168^ to account for certain nontopological shape evolution. This spectral approach, also called persistent Laplacians (PLs),^91,108^ recovers the topological invariants of persistent homology via its harmonic spectra and offers additional nontopological information through its nonharmonic spectra. As a result, it outperforms persistent homology, as shown in a test on more than 30 data sets.^130^ PLs have been extended to many topological domains, such as cellular sheaves,^180^ path complexes,^170^ hypergraphs,^101^ hyperdigraphs,^30^ and directed flag complexes.^199^ An effective software package has been developed for computing persistent topological Laplacians.^81^ This approach was generalized to the Dirac operator in terms of quantum persistence.^3^ Persistent Dirac operators have been considered for simplicial complexes,^3,172^ path complexes,^154^ and Mayer-Dirac operators.^155^ In addition to these topological spectral formulations, persistent Mayer topology was proposed to generalize the chain complex structure in algebraic topology.^139,155^ Moreover, persistent interaction topology^89,90^ has also been proposed for point cloud data. These new formulations extend traditional algebraic topology approaches in data science. Among these new methods, persistent cohomology,^21^ persistent sheaf Laplacians (PSL),^180^ and persistent interaction topology enable local topological analysis.^89,90^ A survey of these approaches is available.^181^ For data on differential manifolds, the evolutionary de Rham-Hodge method^45^ and persistent de Rham-Hodge Laplacians^151^ have been developed. These methods enable manifold topological deep learning (TDL) of biomedical data.^100^ Finally, for one-dimensional curves embedded in three-dimensional space, the multiscale Gauss link integral,^136^ multiscale Jones polynomials,^143^ and persistent Khovanov homology^93^ have been introduced. While the multiscale Gauss link integral can be easily applied, the computation of persistent Khovanov homology for knots, links, and tangles with a large number of crossings remains a challenge.^138,140,141^ Other methodological advances in TDA beyond persistent homology are discussed in more detail in a recent review.^148^

Many data are very complex and cannot be analyzed by using intuitive approaches. Therefore, TDA must be combined with machine learning (ML) to achieve its goals. The first integration of TDA and deep neural networks, called TDL, was introduced by Cang and Wei.^19^ TDL is an emerging paradigm in data science and a new frontier in rational learning.^124^ In the past decade, TDL has been extensively applied in molecular sciences.^125^ Among the most persuasive illustrations that reliably highlight the key benefits of TDL compared to traditional approaches across diverse domains are TDL’s triumphs in the D3R Grand Challenges, a global annual contest focused on computer-aided drug design,^117,119^ the revelation of SARS-CoV-2 evolutionary mechanisms,^40,166^ and the precise predictions of emerging dominant SARS-CoV-2 variants BA.2 ^43^ and BA.5 ^38^ approximately two months ahead. Recent advances in TDL have been reviewed.^124^

Molecular sciences encompass a diverse range of studies, from understanding the structure and behavior of individual molecules, investigating complex interactions within biological systems, to the design of functional materials and the discovery of effective drugs for treating diseases. The wealth of data generated in molecular sciences demands innovative tools that can effectively unravel the hidden patterns and relationships embedded in the information. In the past decade, TDA and TDL have demonstrated numerous successful applications across various domains in various aspects of the molecular sciences. These include macromolecules, drug design and discovery, materials science, and many others. TDA and TDL have accomplished the utmost promise of uncovering hidden patterns, characterizing molecular structures, viral evolution,^38,39,43^ and protein engineering.^129,130^ The review of the early development of TDA, TDL, and other mathematical methods, such as differential geometry and graph theory, for biomolecular systems was given in 2020.^116^

Despite outstanding achievements in TDA applications to molecular sciences, there are obstacles in TDA methodologies that limit their further development. The current TDA-based feature engineering approaches may fail to capture the intricate topology inherent in molecular structures and interactions. Additionally, as the molecular data sets continue to expand in size and complexity, the limitations of conventional analytical methods and current TDA approaches become more apparent. Moreover, AI technologies are evolving rapidly. For example, there have been several groundbreaking achievements in AI and its applications to molecular science, such as AlphaFold.^1,177^ Although there are efforts that explore the use of TDA in the context of AlphaFold and ChatGPT for molecular sciences,^130,167^ it remains an open problem as to how to further position TDA along with rapidly evolving AI technologies. These gaps and challenges call for the integration of cutting-edge AI and new TDA methodologies that can provide a more nuanced and comprehensive understanding of molecular data. To facilitate future development, it is imperative to conduct a timely survey to compile the major TDA approaches with their applications in molecular sciences and to discuss remaining challenges.

In this review, we first review the early research and development of TDA in molecular sciences that have a crucial impact on the current success of TDA and its applications. Thereafter, we highlight the most significant TDA achievements in macromolecules, drug discovery, materials science, and other fields. Finally, we discuss an outlook for new TDA methodologies and their applications in molecular sciences. Through this review, we strive to contribute to the understanding of TDA’s usages, potentials, and challenges in advancing molecular research and inspiring further exploration in this interdisciplinary field. A summary of the applications of TDA in molecular sciences can be found in Figure 1. Since the focus is on TDA and TDL in molecular sciences, many other mathematical approaches, such as curvature-based geometric learning, are not covered.^300,301^

**Figure 1:**
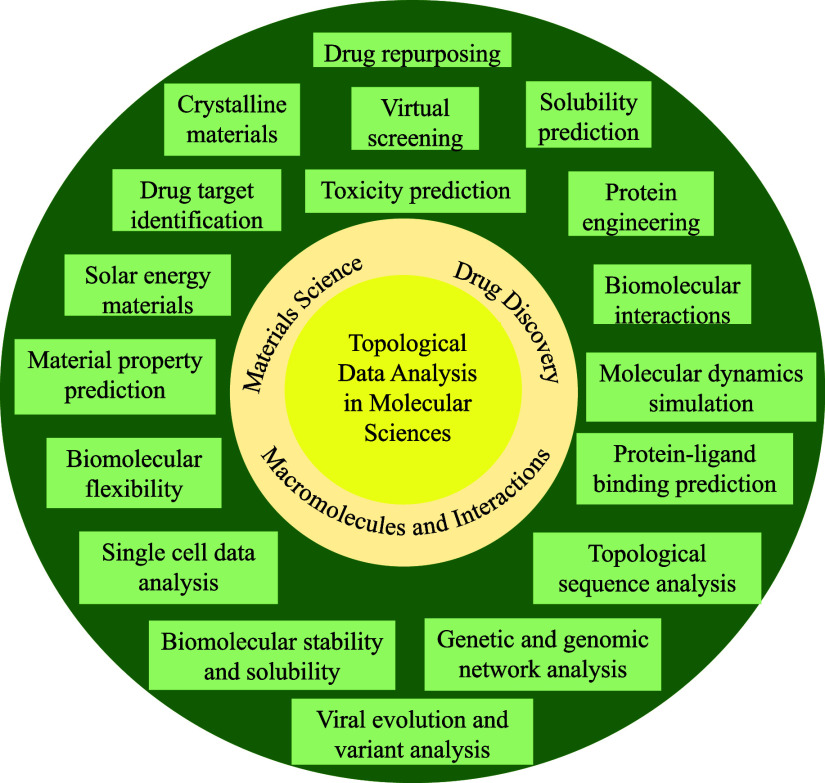
Schematic illustration of the applications of topological data analysis in the molecular sciences.

## 2 Early Research and Development of TDA in Molecular Sciences

### 2.1 Qualitative and Descriptive Analysis

Some of the first applications of topology-related techniques to molecular sciences were qualitative and descriptive, aimed to reveal the biomolecular structure and function relationship. For example, a topology-related tool, alpha complex, was applied to the anatomy of protein pockets and cavities in 1998.^88^ This approach has potential for understanding protein–ligand binding. Another TDA tool, MAPPER, was utilized to analyze the folding of a small RNA tetraloop hairpin in 2008.^11^ The same technique was also applied to the study of biomolecular folding pathways in 2009.^193^

### 2.2 Quantitative and Predictive Analysis

Xia and Wei introduced TDA as a quantitative and predictive tool in 2014.^186^ Persistent homology was, for the first time, introduced for extracting explainable molecular topological fingerprints (MTFs) for linear regression analysis in 2014.^186^ MTFs were employed for protein characterization, identification, and classification. This work offered both all-atom and coarse-grained representations of MTFs to shed light on the optimal cutoff distance in elastic network models for proteins and gave quantitative modeling of protein flexibility, predicting the optimal characteristic distance used in protein B-factor analysis. Finally, MTFs are used to characterize protein topological evolution during protein folding and quantitatively predict protein folding stability. This work reveals the topology–function relationship of proteins.

The Wei team introduced a persistent homology-based method to quantitatively predict fullerene stability.^184^ They discovered that the heat of formation energy correlates with the local hexagonal cavities in small fullerenes, while the total curvature energies of fullerene isomers are linked to their sphericity, as quantified by the lengths of their long-lived Betti-2 bar. Their approach showed strong correlation coefficients between persistent homology predictions and results from quantum or curvature analysis.

One of the first TDA-based ML studies was due to the Wei team in 2015.^15^ In their study, the authors investigated the potential of persistent homology as an independent tool for protein classification. They proposed an MTF-based support vector machine (MTF-SVM) classifier that constructs ML feature vectors exclusively from protein topological fingerprints, which are topological invariants derived during the filtration process. To validate their MTF-SVM approach, they addressed four types of problems. First, they analyze protein-drug binding using the M2 channel protein of the influenza A virus, achieving 96% accuracy in distinguishing drug-bound and unbound M2 channels. Second, they assessed the classification of hemoglobin molecules in their relaxed and taut forms, obtaining approximately 80% accuracy. Third, they performed identification of all-alpha, all-beta, and all-alpha-beta protein domains using 900 proteins, achieving an 85% success rate. Finally, they applied their TDA technique to 55 protein superfamily classification tasks involving 1357 samples and 246 tasks involving 11,944 samples, attaining average accuracies of 82 and 73%, respectively. This study established computational topology as an independent and effective alternative for protein classification.

Gameiro et al. partially elucidated the relationship between a protein’s compressibility and its molecular geometric structure in 2015.^63^ To explore and comprehend the significant topological features of a protein, they modeled its molecule using alpha filtration, which provides multiscale insights into the structure of its tunnels and cavities. By analyzing persistence diagrams, they derived a compressibility measure based on topological features that are indicated to be relevant by the protein’s physical and chemical properties. Their primary finding demonstrates a clear linear correlation between this topological measure and the experimentally determined compressibility for most proteins, where both PDB information and experimental compressibility data are available.

Other early applications of persistent homology to molecular sciences include persistent topology for cryo-EM data analysis in 2015,^188^ and persistent homology analysis of excessively large biomolecular data sets by Xia and Wei.^190^ Persistent homology and dynamical distances were used to analyze protein binding in 2016.^84^

### 2.3 Early TDA Technical Developments for Molecular Sciences

#### 2.3.1 Multidimensional Persistence

In 2015, Xia and Wei introduced multidimensional persistence^187^ and multiresolution persistent homology.^189,190^ They explored persistent homology for simplifying complex biomolecular data, focusing on multidimensional persistence to connect the geometry and topology. They introduced pseudomultidimensional persistence derived from repeated homology filtration on high-dimensional data, like molecular dynamics and multiscale multidimensional persistence, using isotropic and anisotropic scales to form new simplicial complexes. These methods’ utility, robustness, and efficiency are demonstrated in protein folding, flexibility analysis, cryoelectron microscopy denoising, and nanoparticle scale dependence. They observed topological transitions in protein folding and used Laplace–Beltrami flow to distinguish noise from MTFs. Multiscale persistence highlights local Betti-0 features and global Betti-1 and Betti-2 characteristics. These approaches are the early version of persistence images^2^ and are also connected to multiparameter.^148,161^

#### 2.3.2 Object-Oriented Persistent Homology

Earlier applications of persistent homology before 2014 were essentially limited to qualitative data description and analysis. Additionally, persistent homology served as a passive tool for data analysis rather than a proactive one. Wang and Wei introduced object-oriented persistent homology to fill this gap using differential geometry.^163^ A surface free energy functional was defined, and its minimization produced a Laplace–Beltrami operator, creating a multiscale data representation for targeted filtration. This preserved geometric features, enhancing the topological persistence. A cubical complex algorithm was used to update the Laplace–Beltrami flow in the Cartesian representation. Extensive validation confirmed the consistency with Euclidean distance filtration and reliability across mesh sizes. The method analyzed protein and fullerene topologies, predicting fullerene isomer stability via a model linking cavity persistence to the curvature energy. Over 500 fullerene molecules have verified their robustness, offering accurate predictions for ten isomer types. This work connects persistent homology with different geometric analyses and partial differential equation (PDE) modeling.

#### 2.3.3 Element-Specific Persistent Homology

Topology offers maximal abstraction but often loses geometric detail. Persistent homology integrates topology and multiscale analysis to capture topological invariants and connect complex geometry with abstract topology. However, it oversimplifies essential geometric information. Cang and Wei introduced a new method, ESPH or multicomponent persistent homology, to preserve essential biological information during topological simplification in 2017.^17,20^ By partitioning the point cloud into different atom subsets, ESPH can capture the topological features of different atom–atom interactions in proteins. For example, ESPH features generated from carbon atoms are associated with hydrophobic interactions. Similarly, interactions between nitrogen and oxygen atoms correlate to hydrophilic interactions or hydrogen bonds. By combining ESPH with ML, a robust framework for macromolecular analysis was developed to capture physical, chemical, and biological information and interactions in macromolecular systems. For the prediction of protein stability changes upon mutation, this topological approach outperformed the state-of-the-art competing methods.^17^ Further testing on two large datasets showed this ESPH-based machine-learning approach surpasses existing methods in protein–ligand binding affinity predictions.^20^ ESPH uncovered protein–ligand binding mechanisms unattainable by conventional techniques, revealing hydrophobic interactions extending 40Å from the binding site, significantly impacting drug and protein design. The essential idea of ESPH has served as a cornerstone for the TDA of complex data in the past decade.

#### 2.3.4 Topological Deep Learning

TDL, first introduced by Cang and Wei,^19^ is a fast-growing field that leverages topological features to enhance the understanding and development of deep learning models. Due to its explainability, TDL represents a new frontier in relational learning.^124^ By integration of topological concepts, TDL complements graph representation learning and geometric deep learning, making it a promising approach for various ML applications. The original development of TDL, also called TopologyNet, was motivated by the need to predict large data sets in protein–ligand binding affinities and protein stability changes upon mutation. This work also utilized ESPH in feature generation. The element-specific method characterizes different atom–atom interactions instead of directly applying persistent homology to the entire point cloud of a protein–ligand complex. The vectorization of these element-specific features was used in various deep neural networks, including convolutional neural networks (CNNs) and multitask neural networks (MTNNs) for biomolecular property predictions. Due to the interpretability of persistent homology. These methods were some of the first interpretable neural networks (INNs). The results of this approach were state-of-the-art in the field. TDL is a new paradigm in data science and ML.^198^

#### 2.3.5 Multilevel Persistent Homology and Electrostatic Persistence

The Wei team proposed various methods to enhance persistent homology for representing biomolecular data in ML and deep learning applications.^14^ One challenge is that biomolecules and small molecules exhibit multiscale interactions, including electronic, atomic, functional group, residue, domain, intramolecular, and intermolecular scales. These interactions determine the critical physical, chemical, and biological properties. A simplistic filtration approach cannot adequately capture these interactions. To address this, Cang et al. introduced multilevel persistent homology to extract interactions at appropriate spatial scales. Additionally, the authors developed electrostatic persistence to represent, characterize, and describe small molecules and biomolecular complexes. Molecular electrostatics involves the electrostatic forces that govern the structure, function, dynamics, and interactions of molecules, such as ligands, drugs, proteins, and DNA, influenced by charged molecules, amino acids, and the surrounding ionic environment. Electrostatics plays a crucial role in molecular science. Electrostatic persistence captures molecular electrostatics through charge embedding schemes, both utilizing atomic partial charges computed via physical models. Consequently, electrostatic persistence enables the development of physics-informed neural networks (PINNs).

## 3 Macromolecules and Interactions

### 3.1 Biomolecular Stability and Solubility

Biomolecular stability refers to the ability of a biomolecule, a protein, or DNA/RNA to preserve its configuration despite alterations in environmental conditions like temperature, pH, and the composition of the solvent. Solubility indicates the capacity of a biomolecule to remain dissolved in solution under physiological or formulation conditions, critically influencing its stability and bioavailability. Particularly, biomolecular mutations can significantly influence the stability and solubility of a protein or DNA/RNA, which results in altered biomolecular functions and leads to various diseases. During recent decades, TDA has made significant contributions to understanding and predicting biomolecular stability and solubility, providing a unique perspective on complex relationships within biomolecular structures.^186^ By representing biomolecular structures as simplicial complexes, where amino acids are nodes and edges or higher-dimensional simplices represent interactions, such as hydrogen bonds or van der Waals forces, TDA captures the topological features of the biomolecule, offering a more holistic view beyond traditional methods that focus on geometric or energetic considerations. Various early works of TDA have introduced persistent homology for mathematical modeling of biomolecular structures, providing the necessary application of TDA to biomolecular function and dynamics.

In the initial applications of TDA for molecular sciences, Wei and co-workers utilized persistent homology to study the stability of fundamental biomolecular structures, including fullerene, carboranes, and carbon isomers.^34,184^ Their goal was to gain a deeper understanding of how topological fingerprints are being influenced by biomolecular structural changes. For instance, persistent homology has been analyzed on large biomolecular data^187,189,190^ and efficiently resolved ill-posed inverse problems in cryo-EM structure determination.^188^ Further, persistent homology could provide the topological and geometric information to calculate a topological compressibility for proteins, which is well related to the structural stability of proteins.^63^ Persistent homology contributed greatly to protein folding analysis by extracting topological features through the protein unfolding process, allowing researchers to construct models to understand the topology-function relationship between topological features and protein stability.^69,112,160^ Additionally, gradient boosting trees using integrated PL and pretrained transformer-based features were developed to predict mutation-induced protein solubility changes.^173^ These contributions solidified TDA as a robust and powerful tool in the structure analysis of biomolecular data.

### 3.2 Biomolecular Flexibility

The protein *B*-factor, also known as the temperature factor or Debye–Waller factor, quantifies the atomic displacement or structural flexibility within a crystal structure, providing insights into local protein dynamics and conformational stability. Accurate prediction of protein B-factors is an important and meaningful metric in understanding protein structure, flexibility, and function. The TDA-based approaches offer a robust framework for predicting protein *B*-factors by capturing intrinsic structural features and multiscale geometric and topological patterns, enabling noise-resistant characterization of flexibility and dynamic behavior beyond conventional coordinate-based methods.

Mathematically, we need localized models for the *B*-factor predictions. However, persistent homology is global because its topological invariants are typically for the whole data set. Recently, new approaches such as weighted persistent homology,^128^ atom-specific persistent homology,^12^ and evolutionary homology^16^ for protein flexibility analysis. In 2025, Hayes et al. proposed the persistent sheaf Laplacian (PSL), a new effective tool in TDA, to model and analyze protein flexibility.^70^ By representing the local topology and geometry of protein atoms through the multiscale harmonic and nonharmonic spectra of PSLs, the proposed model effectively captures protein flexibility and provides accurate, robust predictions of protein B-factors.

### 3.3 Biomolecular Interactions

Protein–protein interactions (PPIs) are fundamental to cellular signaling, structural organization, and complex formation. TDA made significant contributions to PPI studies by revealing hidden network structures, identifying functional protein modules, and capturing higher-order connectivity patterns compared to traditional graph-based methods.

In the past few years, researchers have developed TDA-based AI models for predicting the changes in PPI binding affinities induced by mutations. The Wei team introduced one of the first TDA-based AI models for predicting the changes of PPI binding affinities induced by mutations TopNetTree,^164^ which integrates persistent homology-based features with a new deep learning algorithm called NetTree that takes advantage of CNNs and gradient-boosting trees. Thereafter, TDA-based AI models that use persistent Hom complex and persistent Tor-algebra were developed and similarly outperformed existing state-of-the-art models in predicting the changes of PPI binding affinities induced by mutations.^98,104^ Persistent homology and molecular dynamics were also employed to investigate amino acid mutation-induced structural changes for PPIs.^83^ Additionally, Wee and Xia proposed persistent spectral (PerSpect) based PPI representation and featurization, and PerSpect-based ensemble learning models for PPI binding affinity prediction.^176^

Unlike the above problems that involve mutation, the direct prediction of protein–protein binding affinity is also important.^137^ Xu et al. recently proposed the persistent Laplacian decision tree (PLD-Tree), a new approach for the direct prediction of protein–protein binding affinities.^191^ This method targets protein chains at binding interfaces, utilizing a PL to capture topological invariants that reflect key interprotein interactions. These topological features are enriched by integrating sequence-based data from evolutionary scale modeling (ESM) derived from a large language model.

PPIs are crucial to biological processes, such as signal transduction, enzymatic regulation, and immune responses, underscoring the importance of precise structural modeling for therapeutic innovation. Dai et al. introduced TopoDockQ, a TDL approach that accurately predicts DockQ scores (p-DockQ) to enhance model selection. Compared with AlphaFold2’s built-in confidence score, TopoDockQ reduces false positives by at least 42% and increases precision by 6.7% across diverse benchmarks.^52^ This advancement expands the possibilities for peptide engineering, particularly in developing therapeutics with customized biochemical properties, enabling a more accurate and flexible peptide design.

### 3.4 Molecular Dynamics Simulation

Molecular dynamics (MD) simulations provide atomistic insights into biomolecular motions and interactions over time. Traditional MD methods often struggle with limited sampling efficiency and difficulty in capturing rare events on biologically relevant time scales. TDA enhances MD by capturing global geometric and topological features, enabling the robust detection of conformational states, transition pathways, and hidden collective dynamics beyond traditional coordinate-based metrics.

The first application of persistent homology to protein folding dynamics was due to Xia and Wei.^186^ These authors studied protein folding and unfolding using MTFs to characterize protein topological evolution during protein folding and quantitatively predict protein folding stability. They found excellent consistency between MTF prediction and molecular dynamics simulation.

Ichinomiya et al. used persistent homology to analyze protein folding.^77,78^ Recently, Arango et al. demonstrated that a persistent homology-based TDA approach, integrated with deep learning, outperformed traditional order parameters by capturing both local and global lipid structural features to robustly predict membrane organization across temperatures.^6^ In 2024, they introduced a persistent homology-based topological learning framework that, combined with attention networks, enabled multiscale prediction of lipid effective temperatures by capturing both local and global structural features beyond traditional metrics.^7^

### 3.5 Protein Engineering

Protein engineering designs and optimizes proteins for desired functions by modifying the sequence, structure, or dynamics. The use of accumulated protein databases and ML models, especially those utilizing natural language processing (NLP), has significantly accelerated the pace of protein engineering in recent years. Advancements in TDA and AI-driven protein structure prediction tools like AlphaFold2 have enabled the development of more potent strategies for protein engineering that are guided by ML and based on the structure. For instance, Xia et al. integrated TDA with graph neural networks and AlphaFold3 for protein complex structure interface quality assessment.^68^

Protein engineering traditionally relies on prior structural and functional knowledge, yet the vast mutational space (20^*N*^) limits exhaustive exploration. While directed evolution offers breakthroughs, experimental scanning remains constrained. TDA overcomes these challenges by capturing global and local structural features in a model-free manner, enabling the efficient navigation of mutational landscapes. By integrating TDA with protein engineering, researchers can identify functionally relevant variants more systematically, thus enhancing the predictive power and accelerating the discovery of proteins with optimized properties.

In recent times, topological AI models have emerged as new approaches to directed evolution and protein engineering. Qiu and Wei integrated PL-based structural features and two auxiliary sequence embeddings with AI to capture mutation-induced topological invariant, shape evolution, and sequence disparity in the protein fitness landscape.^130^ Here, both structure- and sequence-based embeddings are utilized in this ML-based protein engineering approach. It is impressive to see that deeper ML models and emerging large-scale deep mutational scanning databases will continue to enhance the model performance in protein engineering. A review of TDA applications in protein engineering was given by Qiu and Wei.^129^

## 4 Drug Discovery

### 4.1 Drug Target Identification

Drug target identification is fundamental in drug discovery, as it defines the molecular basis of disease intervention, enabling the rational design of therapeutics with higher specificity and efficacy. For instance, Tola et al. identified potential inhibitor compounds for methylcitrate dehydratase by utilizing multiparameter persistence based on persistent homology.^158^ One limitation in the traditional persistent homology used in target identification is that it treats all atoms indiscriminately. Hence, persistent path topology (PPT) was proposed to characterize molecules by incorporating their embedding with element types into their topological analysis.^31^ Furthermore, angle-based filtration PPT is also proposed to complement the existing distance-based filtration PPT. The benefit of PPT allows researchers to handle molecular structures without the need for techniques like element-specific^17^ or persistent cohomology.^21^ With this benefit, Wei and co-workers introduced topological perturbation analysis (TPA) as the inaugural technique of PPT for the analysis of biological networks.^31^

TPA has the unique capability to identify crucial nodes within intricate networks and holds immense potential for applications in various biological networks, such as gene regulatory networks,^55^ PPI networks, signaling networks, metabolic networks, neuronal networks, DNA–DNA–chromatin networks, and transcriptomic networks. This work introduced a fresh perspective to drug target discovery. PPT heralds a new era for future advancements in biological networks, showing promise for drug target identification, discovery of gene motifs, directed evolution, protein engineering, and omics in general.

### 4.2 Virtual Screening

Virtual screening is a computational strategy in drug discovery that systematically evaluates large chemical libraries to identify potential bioactive compounds with specific targets. Traditional approaches often rely on molecular docking or similarity-based methods, which may overlook complex structural or functional relationships. By integrating TDA, virtual screening can capture hidden geometric and topological features of the molecular space, enabling more robust discrimination between active and inactive compounds. This topological perspective enhances predictive power, improves generalization across diverse chemical scaffolds, and facilitates the identification of novel drug candidates beyond conventional chemical similarity boundaries. Additionally, virtual screening through AI learning models significantly reduces the time and cost as compared to traditional drug discovery methods.^82^

The Wei team introduced the first TDL-based virtual screening in 2018.^14^ Their approach achieved great success in the D3R Grand Challenges.^117,119^ Several innovative TDA-based approaches have been proposed to address challenges in identifying novel therapeutics. For example, the topological Laplacian was integrated with AI learning models in the virtual screening of the DrugBank database for hERG blockers.^62^ Zhu et al. built a topology-inferred drug addiction learning model for virtual screening of drug addiction data by integrating multiscale topological Laplacians, deep bidirectional transformer, and ensemble-assisted neural networks.^197^ Additionally, Keller et al. developed a persistent homology-based virtual screening tool for ligand screening.^82^

### 4.3 Protein–Ligand Binding Prediction

The success of TDA has greatly contributed to the field of protein–ligand interactions.^18,95,96^ Protein–ligand interactions are a significant key area to tackle in order to advance drug design and discovery development. One key problem lies in the molecular representation and featurization of protein–ligand interactions, which is important to build successful advanced mathematical AI models.^79,159^ Extracting the important and essential topological and geometric information in molecular structures generates concise and rich topological descriptors in the TDA-based AI models. TDA-based AI models have leveraged algebraic topology, differential geometry, and algebraic graph theory to construct an effective representation of biomolecular systems.^8,14,116^ When integrated with ML or deep learning algorithms, the TDA approach applied in these advanced mathematical models has contributed to tremendous success in protein–ligand binding affinity prediction.^97,105^ Over the years, several TDA-based deep learning models have outperformed numerous existing state-of-the-art traditional molecular descriptor ML models.^103,110,120^

In 2015, the Wei team integrated TDA with ML algorithms for protein classification.^15^ One of the first TDL models developed was in reference,^19^ where TopologyNet was used to predict protein–ligand binding affinities, mutation-induced globular protein folding free energy changes, and mutation-induced membrane protein folding free energy changes.

Recently, a series of tools based on TDA were developed for binding affinities prediction. For example, Feng et al. introduced commutative algebra ML for the affinity predictions of protein–ligand binding and metalloprotein–ligand binding.^61^ Zia et al. presented the persistent-directed flag Laplacian (PDFL), which incorporates directed flag complexes to account for edges with directionality originating from polarization, gene regulation, heterogeneous interactions, etc. They found that the proposed PDFL model outperformed competitors in protein–ligand binding affinity predictions.^199^ Feng et al. developed persistent Mayer homology (PMH) theory based on the standard homology theory, which was validated protein–ligand data sets, including PDBbind-v2007, PDBbind-v2013, and PDBbind-v2016.^60^ Additionally, a series of advanced mathematical methods have been proposed and used on binding affinity data prediction, including persistent Hodge Laplacian learning algorithm,^151^ join persistent homology,^171^ a novel topological ML model (TopoML),^102^ PLDT,^191^ knot data analysis,^136^ multitask-topological Laplacian,^174^ etc.

The noteworthy success of TDA-based deep learning models in the D3R Grand Challenges underscores the effectiveness of this approach in real-world drug design scenarios.^117,119^ These models leverage TDA’s capacity to extract and quantify persistent topological features, allowing for accurate predictions of molecular interactions. The ability of TDA-based AI models to outperform other methods in such competitive settings attests to the practical impact and potential transformative role of TDA in drug discovery.

The emerging advancements in AI have also further accelerated TDA’s contributions in protein–ligand interaction. From NLP to transformer architectures and foundational models like ChatGPT, these emerging models, after pretraining on large-scale and huge amounts of databases with unlabeled data, serve as powerful solutions to boost TDA-based AI models further in protein–ligand interaction. Recently, topological transformers have demonstrated their capabilities by converting 3D protein–ligand complexes into topological sequences, thereby facilitating the application of sophisticated large language models for the analysis of protein–ligand interactions.^26,28^ In particular, the transformer has also demonstrated exceptional efficacy in predicting binding affinity tasks across a range of benchmarks, including those specifically related to SARS-CoV-2.^26^ It evaluated the influence of virus mutations on the effectiveness of drugs, providing vital insight into potential drug resistance. Additionally, Chen et al. presented a topological transformer integrating a persistent topological hyperdigraph Laplacian and transformer models for protein–ligand interaction predictions.^29^

### 4.4 Drug Repurposing

Another important area in drug discovery is topological AI-based drug repurposing, where topological AI models were developed to identify existing drugs repurposed for drug addiction treatment.^197^ This can significantly reduce the drug development process, as existing drugs have already undergone extensive safety testing. In particular, antibiotic drug discovery has also garnered huge interest in drug discovery communities, as topological AI models can also be developed to identify existing drugs for antibiotic resistance.^157^ This study applied a topological structure–activity data analysis model to repurpose FDA-approved drugs as candidate antimicrobials against *Escherichia coli*. By comparing topological signatures of drug–target interactions and *E. coli* proteins, the approach recovered known antibiotics and nominated diverse drug classes (e.g., antitumor agents, antihistamines, and hypoglycemics) as potential antimicrobials. Cross-species topological similarities implied broader spectrum activity and novel targets. This work demonstrated TDA-driven virtual screening as a rapid, mathematically grounded strategy for drug repurposing to address antimicrobial resistance.

Furthermore, in 2024, Du et al. proposed a TDA-enhanced method, persistent spectral theory for topological differentiation of PPI networks from differentially expressed gene data and identified three pivotal molecular targets for antiaddiction drug repurposing from DrugBank.^55^ Using topological Laplacians and algebraic-graph embeddings, Feng and Wei applied TDA-integrated ML to screen DrugBank for hERG cardiotoxicity, identifying 227 potential blockers out of the 8641 DrugBank compounds and demonstrating TDA’s utility for safer drug repurposing.^62^

Cottrell et al. present a sequential model for selecting target–drug pairs for drug repurposing aimed at Alzheimer’s disease (AD) targets, derived from population-level single-nucleus RNA sequencing (snRNA-seq) studies of AD progression in microglia and various cell types from an AD-affected brain vascular tissue atlas, encompassing hundreds of thousands of nuclei across multiple patients and brain regions.^51^ They employ PSL^180^ to analyze PPI networks of AD targets identified through differential gene expression (DEG) and apply ML models to predict repurposable DrugBank compounds for molecular targeting.

### 4.5 Solubility Prediction

Solubility prediction plays an important role in drug discovery by enabling early assessment of drug candidates’ developability, guiding lead optimization, and reducing late-stage attrition due to poor pharmacokinetic properties. The prediction accuracy depends crucially on molecular descriptors, which are typically derived from a theoretical understanding of the chemistry and physics of small molecules. Integrating the TDA-based method with the ML algorithm has contributed to good prediction of aqueous solubility with comprehensive molecular representation.

In 2018, Wei and co-workers proposed element-specific persistent homology combined with multitask deep neural networks for simultaneous predictions of partition coefficient and aqueous solubility, which provided multiscale and multicomponent topological invariants to describe the molecular properties and achieved some of the most accurate predictions of aqueous solubility.^183^ Additionally, Ehiro generated molecular descriptors from the Morgan fingerprint using persistent homology and improved the prediction accuracy on solvation free energy and water solubility data sets.^58^ Dong et al. proposed a modality-adaptive method based on an improved multiobjective optimization algorithm for molecular property prediction, including water solubility, which integrated TDA, the teacher learning mechanism, and graph centrality measures.^53^

### 4.6 Toxicity Prediction

Toxicity prediction on drug candidates is one of the key procedures of drug discovery. TDA acts as a transformative tool in small molecular data challenges of toxicity data sets.^54^ TDA combined with deep learning approaches can offer valuable insights into toxicity prediction. The ability of TDA to discern complex and subtle patterns in molecular data enhances the accuracy of the toxicity predictions, providing a holistic view of the underlying mechanisms. This innovative approach in toxicity prediction not only aids in the early identification of potentially harmful compounds but also contributes to the development of safer and more effective drugs through a deeper comprehension of molecular interactions and their implications for toxicity.

In order to enhance the prediction of small quantitative toxicity data sets, Wu and Wei developed multitask deep learning models to integrate with TDA for learning multiple toxicity tasks simultaneously and exploit commonalities as well as differences across different tasks.^182^ The integration of algebraic graph representations and bidirectional transformer-based embeddings with a variety of ML algorithms, including decision trees, multitask learning, and deep neural networks, has produced great performance across eight molecular data sets, involving quantitative toxicity data sets.^25^ Recently, Rong et al. proposed a topological fusion network leveraging TDA to capture multiscale topological features and their method outperformed the state-of-the-art method by 2.4% on the ClinTox data set for the classification task.^133^ Other TDA-based methods have been developed for toxicity predictions, including nanoparticle toxicology assessments.^122^

## 5 Materials Science

### 5.1 Crystalline Materials

Progress in the field of materials science is often gradual and demanding, posing a significant challenge in keeping up with the growing need for material characterization. When dealing with intricate data sets in crystalline materials, a crucial challenge arises in determining methods to create low-dimensional representations for input crystal structures that consist of valuable chemical insights. One of the early applications involves the use of the topological concept to analyze defects in crystal structures.^67,73,147^ Persistent homology was also applied to lithium cluster structure prediction^46^ and two-dimensional network analysis.^114^

In recent years, high-throughput computational methods, such as density functional theory (DFT), have been employed to predict the properties of both experimental and hypothetical inorganic compounds. Although experimental and computer simulation methods have contributed to a vast amount of high-quality open databases, such methods still lack efficiency and remain expensive for heavier elements, strongly correlated electrons, and large molecules. Furthermore, DFT is not well-suited for large and diverse material data sets. As such, TDA-based descriptors have been proposed to serve as powerful tools for the high-throughput screening of materials with specific topological features. These descriptors enable the identification of materials with the desired properties, guiding researchers in the design and discovery of novel crystalline materials. For instance, atom-specific persistent homology (ASPH)-based ML models were developed and proved to achieve highly accurate predictions of DFT-calculated formation energy.^80^

Comprising metal ions or clusters linked to organic ligands, metal–organic frameworks (MOFs) represent a distinct category of crystalline materials that self-assemble into porous structures with exceptional tunability. In 2021, persistent homology was applied to the embeddings of MOFs.^86^ It automatically encapsulates geometric and chemical information directly from the material system. In 2025, Chen et al. introduced a TDA-based category-specific topological learning for robust material property prediction.^24^ More recently, they have developed an interaction topological Transformer for multiscale learning in porous materials.^27^ In 2023, Shekhar and Chowdhury introduced a feature-based representation of materials using tools from TDA for the prediction of hydrogen storage in MOFs.^135^ Additionally, Yang et al. developed a topological descriptor based on TDA, which was combined with the extreme gradient boosting algorithm to predict the adsorption performance of MOFs.^192^

### 5.2 Solar Energy Materials

Recently, materials such as organic–inorganic halide perovskites (OIHPs) have gained significant attention in the field of solar cells due to their remarkable cost-effective photovoltaic capabilities, placing them in competition with widely used silicon solar cells. Although OIHP solar cells have remarkable performance, many high-efficiency OIHP solar cells contain lead and face challenges due to their poor stability in an ambient environment. Consequently, this sparks an urgent interest in discovering new perovskite variants with enhanced properties to overcome these limitations.

In 2022, TDA-based approaches such as persistent homology and persistent Ricci curvature were integrated with ML to better understand and predict the formation energy and bandgap values of OIHP materials.^5^ Formation energy and bandgap are important material properties for understanding the conductive and insulating behavior of OIHP materials, crucial for optimizing their performance in solar cells. Persistent homology and persistent Ricci-curvature-based descriptors also demonstrated effective classification over different OIHP crystal configurations and different atom types. Additionally, persistent homology was applied to classify high-entropy alloy data sets containing body-centered cubic (BCC) and face-centered cubic (FCC) crystal structures.^145^

### 5.3 Material Property Prediction

Understanding the structure–function relationships in materials is the key point of material discovery. Compared with traditional experimental approaches, TDA-based methods present their advantages in computational efficiency, uncovering structure–property correlations, and providing physical insights into material behavior.^196^ For instance, lithium superionic conductors (LSICs) are vital for next-gen solid-state batteries but hard to discover due to vast chemical space, limited data, and complex structure–function links. In 2025, Chen et al. presented a TDA-based multiscale topological learning framework integrating algebraic topology and unsupervised learning, extracting features, and using screening metrics. They found 14 new LSICs, accelerating LSIC identification and aiding material discovery.^32^

Enhancing the accuracy of energy predictions in multilithium-atom systems is essential for optimizing atom properties and benefiting material design. Chen et al. explored the application of persistent topological Laplacian, a TDA-based method to effectively capture the intrinsic properties of many-body interactions, accelerating the discovery of new materials and enhancing the efficiency of material development.^33^ They also introduced a TDA-based framework for extracting structural features of materials and achieved up to 55% reduction in prediction error for defect-sensitive properties.^162^

Simulating how molecular building blocks self-assemble into functional complexes constitutes a key research focus in materials science. Spirandelli et al. presented a long-range topological potential, measured through weighted total persistence, which they integrated with the morphometric approach to solvation-free energy.^146^ Additionally, a persistent multicover method was introduced for polymer property prediction, integrating with the gradient boosting tree algorithm.^194^

### 5.4 Other Materials

Persistent homology has also been applied to many other materials studies. The Wei team applied persistent homology to the analysis of nanostructures in 2015.^184^ Since then, this approach has been applied to nanoporous materials,^59,85^ polymeric materials,^71^ and soft matter.^87,107,142^ Persistent homology was applied to give a better understanding of the thermal stability,^47^ diffraction patterns,^123^ hierarchical structure,^72,111^ medium-range order of amorphous materials.^115,144^ For example, it was used to extract topological features from the microphase-separated structures of polymeric materials, which were successfully utilized to predict proton conductivity of polymeric materials.^71^

Persistent homology was combined with molecular dynamics to analyze the changes in topological information on liquid structures in ref 134. Additionally, persistent homology-based ML models were also developed for lithium cluster structure prediction.^46^ Software tools like HomCloud have been developed specifically for researchers who are interested in applying persistent homology and inverse analysis in the field of materials research.^121^

Recently, the theory of path homology has been employed to understand the role of mirror-symmetric sublattices that obstruct the creation of periodic unit cells in amorphous materials.^31^ A concise review which encapsulates the developments of TDA-based ML models for material sciences is detailed in ref 195.

Additionally, representations of energy landscapes by sublevel-set persistent homology were presented with *n*-alkanes as an example.^113^ The shape of data and molecular representation in chemistry were discussed in relation to persistent homology analysis.^10,159^

## 6 Other Applications in Molecular Sciences

### 6.1 Topological Sequence Analysis

Sequence data, encompassing DNA, RNA, and protein sequences, possess complex, multiscale structures that present considerable challenges to conventional analysis methods that rely on alignment or purely statistical representations. While TDA has achieved increasing success in capturing the global features of complex data,^8,23^ its application to sequence data in biology is still relatively underexplored.

Hozumi and Wei introduced a k-mer topology approach for topological sequence analysis (TSA) of genomes.^75^ This method utilizes persistent homology and/or PL to characterize genome sequences for variant detection, species classification, and phylogenetic tree analysis. Topological genetic distance and topological antigenic distance were defined. TSA outperforms state-of-the-art methods for phylogenetic and antigenic analysis. Recently, Liu et al. proposed two approaches to generalize the TDA of genomes. One approach is category theory-based TSA, which treats a sequence as a resolution category, capturing its hierarchical structure through a categorical construction.^94^ The other is based on Δcomplex by constructing Δ-complexes and classifying spaces, generating persistent homology, and persistent path homology on genome sequences.^92^ These methods all had good performance across a variety of tasks, including the phylogenetic analysis of SARS-CoV-2 variants, prediction of protein-nucleic acid binding affinities, and biological clustering. Suwayyid et al. introduced commutative algebra k-mer learning for genomic sequence analysis, which bridges between commutative algebra, algebraic topology, combinatorics, and ML to establish a new mathematical paradigm for comparative genomic analysis.^153^

Identifying novel and functional RNA structures remains a significant challenge in RNA motif design and is critical for advancing RNA-based therapeutics. A computational, topology-based approach combined with unsupervised machine-learning algorithms was used to estimate the size and content of the database of RNA-like graph topologies.^169^ This work provides valuable insights into the scope of the RNA motif universe and informs RNA design strategies, offering a new framework for predicting RNA graph topologies and guiding the discovery of novel RNA motifs—potentially enabling the development of antiviral therapeutics through subgraph assembly.

### 6.2 Genetic and Genomic Network Analysis

Genetic and genomic network analysis explores interactions among genes, regulatory elements, and molecular pathways to reveal system-level biological mechanisms. Traditional methods often struggle with high-dimensional noise, nonlinear dependencies, and incomplete representation of complex biological interactions. TDA-based approaches provide advantages by characterizing high-dimensional, nonlinear, and multiscale structural patterns, enabling robust identification of hidden modules and network dynamics beyond conventional graph-based methods.^131^

Recently, Ramos et al. presented a novel method using persistent homology to analyze the role of driver genes in higher-order structures within Cancer Consensus Networks derived from main cellular pathways, which provided an approach to distinguish drivers and cancer-associated genes from passenger genes.^132^ Masoomy et al. applied persistent homology to cancer gene networks, revealing distinct topological deviations, with loops dominating cancer cells and voids prevalent in healthy cells, highlighting higher-order structural differences.^106^ Duman and Pirim employed persistent homology on *Arabidopsis* weighted gene coexpression networks from microarray data, using bottleneck distances and clustering to distinguish stress responses, demonstrating efficient detection of shared topological features.^56^ Additionally, Platt et al. used persistent homology to identify phenotypes that tended to be dominated by metabolic syndrome descriptions.^126^

### 6.3 Single-Cell Data Analysis

Single-cell data are highly dimensional and sparse, reflecting cellular heterogeneity, rare populations, and dynamic transcriptional states at single-cell resolution. TDA offers a powerful framework for single-cell transcriptomics by capturing high-dimensional, nonlinear structures beyond traditional clustering.^76^ Unlike linear methods, TDA preserves global and local topological features, enabling the robust identification of rare cell populations, continuous differentiation trajectories, and hidden functional relationships within complex cellular heterogeneity. For instance, the Hodge decomposition method has been developed and successfully applied to RNA velocity field, which captures the cell dynamic information in the biological processes^149,150,152^

Currently, a series of topologically enhanced approaches has been proposed to enhance existing approaches in single-cell data analysis. For instance, PL-enhanced PCA was proposed to tackle multiscale and multiclass heterogeneity problems in single-cell data.^48,49^ A k-nearest neighbor (kNN) PL technique was also introduced to improve upon the traditional persistent homology. Unlike traditional persistent homology, which filtrates by varying a distance threshold, kNN-PL filtrates by varying the number of neighbors in a kNN network at each step. These techniques were successfully applied to microarray and single-cell RNA sequencing data.^48,49^ Thereafter, methods such as topological nonnegative matrix factorization (TNMF) and robust topological nonnegative matrix factorization (rTNMF) were also introduced and outperform all other NMF-based methods in single-cell RNA sequence analysis.^74^ Both TNMF and rTNMF are PL regularized NMF methods that can better capture multiscale geometric information and improve performance compared to other NMF-based methods.

Recently, Cottrell and Wei proposed multiscale cell–cell interactive spatial transcriptomics analysis. This approach integrates the advantages of an ensemble of multiscale topological representations of cell–cell interactions in the gene expression space with those of advanced spatial deep learning techniques.^50^

### 6.4 Viral Evolution and Variant Analysis

Viral evolution involves genetic mutation, selection, and adaptation that drives viral diversity and pathogenicity. The Severe Acute Respiratory Syndrome Coronavirus 2 (SARS-CoV-2) caused a global COVID-19 pandemic since late 2019, which evolved into numerous variants that have led to several waves of COVID-19 infections. SARS-CoV-2 evolves through mutations to improve its evolutionary adaptability. The Wei team has utilized TDA to advance the study of viral evolution by uncovering evolutionary mechanisms, predicting emerging dominant variants, and capturing high-dimensional genomic patterns beyond traditional phylogenetic methods.^40^ Additionally, TDA-based methods and mathematical AI offer an accurate and efficient alternative to experimental determination of viral infectivity, vaccines, and antibody resistance^42^ via computing binding free energy changes after mutation.^40,99^

For example, the Wei team applied TDA to model the interactions between COVID-19 spike protein in the receptor-binding domain (RBD) and the host’s angiotensin-converting enzyme 2 (ACE2).^178^ Using in silico deep mutational scanning, they predicted two mutation sites 452 and 501 on the viral spike in summer 2020,^40^ which were subsequently found to host the key mutations of all prevailing variants, Alpha, Beta, Gamma, Delta, Theta, Mu, and Omicron, BA.2, BA.4, and BA.5, etc. Additionally, a SARS-CoV-2 evolution mechanism, natural selection via mutation-induced infectivity strengthening, was discovered in summer 2020.^40^ This discovery was achieved through the integration of silico deep mutational scanning, genotyping of viral genomes extracted from the world population, AI, and computational biophysics, i.e., mutation-induced protein–protein binding affinity changes. Another SARS-CoV-2 evolution mechanism, natural selection via antibody resistance or vaccine breakthrough, was first revealed in late 2021 by the team.^166^ This mechanism was discovered with the TDL-predicted mutational disruptions of 135 antibody-spike binding complexes and the correlation between virus mutations and the vaccination rate in many countries. Based on two mechanisms, they predicted emerging dominance of Omicron BA.2 in early February 2022,^43^ that was confirmed by the World Health Organization (WHO) in late March 2022. They further predicted the emerging dominance of Omicron BA.4 and BA.5 on May 1, 2022,^38^ which was confirmed by the WHO in early July 2022.

Technically, topological AI models in protein–ligand interactions have also been extended to study drug target interactions with SARS-CoV-2 main protease and drug resistance with Pfizer’s drug PAXLOVID.^26,118^ A review about SARS-CoV-2 modeling, simulations, and predictions focused on its molecular-level methodologies from the aspects of biophysics, mathematical approaches, and ML, including deep learning, bioinformatics, and cheminformatics in the applications of SARS-CoV-2 is detailed in ref 64. Wee and Wei proposed an AlphaFold3-assisted multitask-topological Laplacian model to enhance the prediction of deep mutational scanning and binding free energy changes upon virus mutations.^175^

Topological AI models such as TopNetmAb were developed to predict the binding free energy changes for SARS-CoV-2 RBD-ACE2 and RBD-antibody complexes due to RBD mutations.^9,36,37^ TopNetmAb is also used to analyze how the RBD mutations on the Omicron variant affect the viral infectivity and efficacy of existing vaccines and antibody drugs.^43^

Another TDA-based AI model, TopNetTree, was developed in the study of viral mutation.^41^ Apart from the 8338 PPI entries that TopNetTree is trained on, more training data related to SARS-CoV-2 were incorporated in order to improve the overall model performance. TopNetTree was also used to analyze the SARS-CoV-2 mutations in the United States, which revealed that two of four SARS-CoV-2 substrains in the United States became potentially more infectious.^165^ TopNetTree and TopNetmAb are applied in antibody studies, which allowed researchers to design and analyze COVID-19 antibody candidates by integrating TDA and AI.^35^

Extending from persistent homology, a new model TopLapNetGBT which integrates PL with deep learning, improves the performance for predicting mutation-induced PPI binding free energy changes.^38^ More importantly, this model successfully predicted Omicron BA.4 and BA.5 as dominant variants before the WHO officially announced in June 2022. Similarly, TopLapNet was applied to determine emerging dominant SARS-CoV-2 variants in 2022.^39^ Additionally, Chen et al. proposed a TDL paradigm to facilitate in silico deep mutational scanning.^44^ Persistent topological Laplacians were proposed to study the protein structures of the SARS-CoV-2 spike receptor binding domain.^179^

## 7 Outlook

TDA holds immense potential to revolutionize molecular sciences. Over the past decade, TDA has transformed the study of macromolecules, molecular interactions, drug design and discovery, and materials science. To fully realize this potential and accelerate TDA’s expansion into other areas of molecular sciences, the research community must maintain an interdisciplinary approach rooted in innovation, collaboration, and open science principles. This approach requires the integration of expertise from mathematics, biology, chemistry, materials science, physics, computer science, and ML to foster a dynamic environment for cross-disciplinary collaboration. By encouraging open communication and idea-sharing, TDA can evolve rapidly, overcome current limitations, and drive groundbreaking discoveries.

The achievements of TDA in molecular sciences highlighted some areas in which TDA can be further improved to benefit molecular sciences. The recently proposed interaction homotopy and interaction homology can be applied in protein–ligand interactions, protein–protein interactions, drug–target interactions, and antibody–antigen interactions.^89^ The mathematical construction of interaction homology would allow researchers to have a better understanding of interactions.

Although TPA has been successful in detecting important nodes within various complex networks,^31^ TPA should be further explored in order to design the localized version of TPA, which could enable precise detection of functional modules within complex biological networks.

Additionally, the applications of persistent sheaf analysis^180^ and weighted persistent homology should be further explored. For example, these concepts have huge potential for the TDA community to design new hypergraph embeddings that can assign weights or emphasize molecular functional groups such as benzenes, esters, thiols, amines, etc.

Moreover, differential topology^151^ and geometric topology^138^ are expected to play a more important role in molecular sciences. The development of effective computational tools for these approaches is needed.

Further, although commutative algebra is a distinguished field, it is deeply connected to algebraic topology. The persistent commutative algebra introduced by Suwayyid and Wei enables the use of graded Betti numbers for molecular characterization.^156^ This new field is open for further in-depth exploration.

Furthermore, the integration of TDA, quantum computing, and AI for molecular sciences is a promising new direction.

Finally, initiated by Cang and Wei,^19^ TDL has become a new frontier in rational learning.^124^ There is great potential to further explore this paradigm in molecular sciences, particularly through large language models (LLMs), foundation models, artificial general intelligence (AGI), the model context protocol (MCP), and other advanced AI platforms.
